# Characterizing dynamics of serum creatinine and creatinine clearance in extremely low birth weight neonates during the first 6 weeks of life

**DOI:** 10.1007/s00467-020-04749-3

**Published:** 2020-09-17

**Authors:** Tamara van Donge, Karel Allegaert, Verena Gotta, Anne Smits, Elena Levtchenko, Djalila Mekahli, John van den Anker, Marc Pfister

**Affiliations:** 1 Pediatric Pharmacology and Pharmacometrics, University Children’s Hospital Basel (UKBB), University of Basel, Basel, Switzerland; 2 Department of Development and Regeneration, KU Leuven, Leuven, Belgium; 3 Department of Pharmaceutical and Pharmacological Sciences, KU Leuven, Leuven, Belgium; 4 Department of Clinical Pharmacy, Erasmus MC, Rotterdam, The Netherlands; 5 Neonatal Intensive Care Unit, University Hospitals Leuven, Leuven, Belgium; 6 Department of Pediatric Nephrology and Organ Transplantation, University Hospitals Leuven, Leuven, Belgium; 7 Division of Clinical Pharmacology, Children’s National Health Hospital, Washington, DC USA; 8 Intensive Care and Department of Pediatric Surgery, Erasmus MC Sophia Children’s Hospital, Rotterdam, The Netherlands

**Keywords:** Reference ranges, Glomerular filtration rate, Creatinine reabsorption, ELBW neonates, Model-based simulations

## Abstract

**Background:**

Characterizing the dynamics of serum creatinine concentrations (Scr) and associated creatinine clearance (CLcr) as a measure of kidney function in extremely low birth weight (≤ 1000 g; ELBW) neonates remains challenging.

**Methods:**

We performed a retrospective study that included longitudinal Scr (enzymatic assay) data from 148 ELBW neonates up to 6 weeks after birth. Change of Scr and inter-individual variability was characterized with nonlinear mixed-effect modeling. Key covariates such as gestational age (GA), mode of delivery (MOD), and treatment with ibuprofen or inotropic agents were investigated.

**Results:**

A total of 2814 Scr concentrations were analyzed. GA was associated with Scr at birth (higher with advancing GA), and GA and MOD showed an association with postnatal maturation of CLcr (faster clearance increase with advancing GA and after C-section). Small CLcr decrease (≤ 5%) was quantified during ibuprofen treatment. For a GA of 27 weeks, mean Scr (estimated CLcr) at birth was 0.61 mg/dl (0.23 ml/min), increasing to 0.87 mg/dl (0.27 ml/min) at day three, and decreasing to 0.36 mg/dl (0.67 ml/min) at day 42 after birth.

**Conclusions:**

We report the first mathematical model able to characterize Scr and CLcr in ELBW neonates during the first 6 weeks of life in a quantitative manner as a function of GA, MOD, and ibuprofen treatment. This model allows the derivation of GA-adjusted reference ranges for ELBW neonates and provides a rationale for normative Scr concentrations, and as such will help clinicians to further optimize monitoring and treatment decisions in this vulnerable patient population.

**Electronic supplementary material:**

The online version of this article (10.1007/s00467-020-04749-3) contains supplementary material, which is available to authorized users.

## Introduction

The availability of reference ranges for any specific laboratory test or biomarker to support clinical decision-making and tailor therapy to the individual patient will greatly support neonatal care [1, 2]. Variability is a key feature in the neonatal population since maturational physiological changes are most prominent during early infancy. This variability is related not only to differences in current weight, gestational age (GA), or postnatal age (PNA), but also to morbidities, co-medication, or nutritional and fluid management. Throughout the neonatal period, serum creatinine (Scr) concentrations in preterm neonates vary tremendously due to this large inter- and intra-individual variability [3, 4].

Despite its limitations, Scr is a commonly measured and easily accessible biomarker to estimate glomerular filtration rate (GFR). The clearance of Scr defines the volume of blood plasma that is cleared of creatinine per unit time and approximates the GFR. Many mathematical equations have been proposed to reflect creatinine clearance or estimated GFR in newborn infants, mostly by means of linear regression methods [4–8]. In classical linear regression, there is only one level of unexplained variability (difference between observation and predicted value). In contrast, applying modeling and simulation techniques rather than classical linear regression methods allows describing and quantifying the maturation processes underlying neonatal physiology with multiple levels of variability. These population models are characterized by the typical individual (the mean) and the random effects, describing the variability of the data [9]. These random effects are divided into two levels: the difference between individuals (inter-individual variability, IIV) and the difference between the individual prediction and the observation (individual prediction error, comprising also analytical imprecision of the observation).

Although the developed equations have supported clinicians in the assessment of estimated GFR, the physiology and normative range of Scr in extreme preterm infants are still not fully understood and the accurate assessment of kidney function remains challenging. Until now, we are not aware of any mathematical modeling effort applying modeling and simulation techniques to Scr data in neonates.

Traditionally, the dogma has been to ignore elevated Scr in preterm infants during the first days after birth as they are considered uninformative about creatinine clearance due to maternal Scr transfer. Additionally, passive reabsorption across immature leaky kidney tubules contributes to transient accumulation of creatinine during the first days of life, which suggests that part of elevated Scr after birth may still reflect neonatal and not maternal kidney function [10].

Determining and establishing (gestational) age-dependent Scr reference ranges early in neonatal life could support the clinical evaluation of kidney function, especially when considering extremely low birth weight neonates (ELBW; ≤ 1000 g). The aims of this study were (i) to characterize the dynamics of Scr and associated creatinine clearance during the first 6 weeks of life by the development of a mathematical model, (ii) to identify and quantify factors influencing Scr and kidney function in this specific population, and (iii) to derive age-dependent Scr reference ranges to facilitate monitoring and evaluation of kidney function in ELBW neonates.

## Methods

### Study population and clinical characteristics

Data for this retrospective study is based on longitudinal Scr data of neonates admitted to the neonatal intensive care unit (NICU) of the University Hospitals Leuven. The study population used for model development has been previously described and includes all neonates admitted to the NICU of the University Hospitals Leuven between July 2007 and August 2011 whose Scr concentrations were available [11]. For the current analysis, all Scr concentrations during the first 6 weeks of life (extended to all available Scr data in the first 42 days after birth instead of the more narrow time windows in the initial paper [3]) were retrieved from these ELBW neonates (≤ 1000 g). In addition, all clinical data (demographic characteristics and information on co-medication) were retrieved from medical files, verified, and complemented. Detailed information on the perinatal characteristics (i.e. 21% of women were diagnosed with pre-eclampsia, 33% of ELBW neonates were small for gestational age, median duration of ventilation was 8 days, and median days until full enteral feeding was 34 days) can be found in the initial paper, published in this journal [3]. Clinical data, such as GA, birth weight, current weight, sex, length, mode of delivery (MOD), ibuprofen treatment (IBU), treatment with inotropic agents (as disease severity marker), maternal betamethasone treatment, neonatal death, and Scr concentrations from birth through 6 weeks of life were retrieved. Patients were excluded if there was no information on GA and/or MOD or if they were not classified as ELBW.

An external evaluation analysis was performed based on an additional dataset of a retrospective study to explore postnatal albuminemia trends. This dataset includes all neonates admitted to the NICU of the University Hospitals Leuven between June 2015 and March 2017 whose albuminemia data were available. For the external evaluation dataset, ELBW cases were selected and Scr measurements were extended to the first 6 weeks of life for the current study. Ethical approval of the current study covered the additional data search for both datasets (model development and evaluation, S63405). Scr was analyzed enzymatically by Roche Cobas c702 (Roche Diagnostics, Mannheim, Germany) in both datasets, and all measurements were isotope dilution mass spectrometry (IDMS) traceable.

### Model development

Change of Scr during the first weeks of life was characterized by the development of mathematical turn-over model, in which Scr concentrations (mg/dl) are described as a result of creatinine production (mg/day) and time-varying creatinine clearance (L/day). Respective mean population parameters and inter-individual variability (IIV) were obtained by nonlinear mixed-effects modeling analysis (Online Resource 1). Lognormal parameter distributions within the study population were assumed, and a proportional error model was used to characterize the residual variability.

Distribution of Scr was assumed to reflect the total body water, similar as for aminoglycosides. Studies on gentamicin and amikacin disposition in (preterm) neonates observed volumes of distribution (Vds) between 0.3 and 0.8 L/kg [12, 13]. Based on this evidence, Vd of creatinine was set at 0.7 L/kg for our study population [14, 15]. Sensitivity analyses were performed to investigate the impact of ranging Vds (0.3–0.8 L/kg) on the estimated population parameters, and additional sensitivity analysis was carried out to compare Vd set at 0.7 L/kg and the formula to calculate total body water suggested by Shaffer et al. [14]. To include the physiological and crucial weight changes during the neonatal period, Vd was based on linear interpolation between birth weight and current weight measurements. If no current weight measurements were collected, birth weight was used to determine Vd. Body surface area (BSA) was determined with the equation of Ahn and was used to convert clearance estimates to ml/min/1.73 m^2^ (Eq. 1) [16].

1 $$ \mathrm{BSA}\ \left({\mathrm{m}}^2\right)=\frac{10.602\times {{\mathrm{weight}}_{\left(\mathrm{g}\right)}}^{0.6561}}{10000} $$

Key covariates such as GA, MOD, birth weight, current weight, birth length, sex, and treatment with ibuprofen or inotropic drugs were investigated applying a stepwise forward selection and backward deletion approach based on the likelihood ratio test (*p* < 0.05). Model evaluation was performed by predefined selection criteria, such as the precision of the estimated parameters (residual standard error, RSE), the maximization of the likelihood (decrease of objective function value of at least 3.84 points for one additional model parameter in nested models), goodness-of-fit plots (observed versus predicted creatinine concentrations), and visual predictive checks. Software package Monolix (version 2019R1. Antony, France: Lixoft SAS, 2020, http://lixoft.com/products/monolix/) was used to fit individual data to the mathematical model. Data handling, graphical visualization, and numerical calculations were performed in R (version 3.5.1; R Development Core Team, Vienna, Austria, http://r-project.org).

### Model evaluation

An external evaluation was performed to evaluate the predictive performance of the developed creatinine model. The mean percentage error (MPE) and relative MPE (RMPE) were calculated as measures of bias, and the mean squared error (MSE) and relative root mean squared error (RMSE) were calculated as measures of precision (Online Resource 1) [17].

### Development of Scr reference ranges: model-based simulations

The developed mathematical model incorporating covariates was leveraged to simulate Scr concentrations and creatinine clearance values for typical ELBW neonates stratified for three reference GA values, namely 24, 27, and 32 weeks. In a deterministic simulation (no IIV), predicted median profiles were illustrated as a function of included covariates. A stochastic simulation that includes IIV was performed in order to obtain reference ranges, defined as 95% percentiles, for Scr concentrations and creatinine clearance values. In total, 1000 simulations were performed for each reference GA value.

## Results

Data from 158 ELBW neonates were collected, of whom ten qualified as dropouts (no information on GA or MOD) and were excluded from the analysis. Over a period of 6 weeks after birth, a total of 2814 Scr concentrations of 148 ELBW neonates were included in the model development analysis. Sixty-nine ELBW neonates were included in the external evaluation analysis with 1212 Scr concentrations.

### Study population and clinical characteristics

**Fig. 1:**
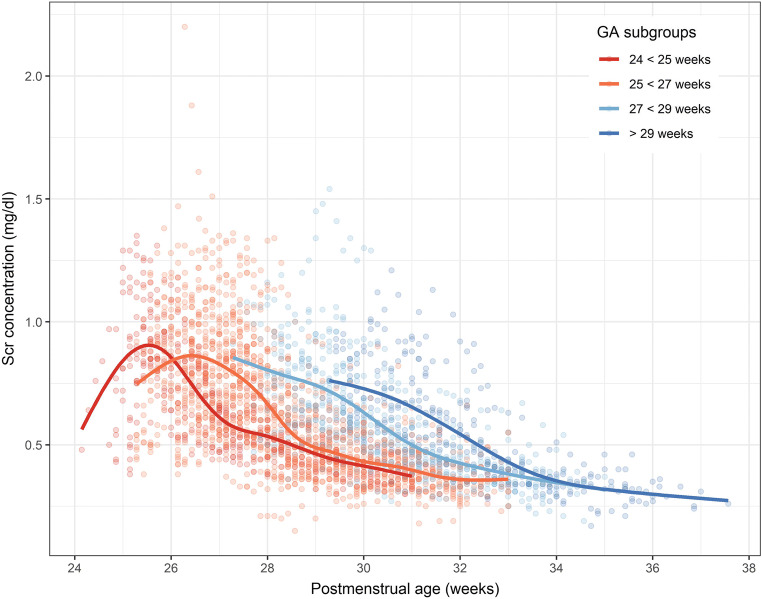
Observed serum creatinine (Scr) concentrations versus postmenstrual age (gestational age (GA) + postnatal age), grouped per GA category

An overview of observed individual Scr concentrations versus postmenstrual age is presented in Fig. 1. Our population qualified as extremely preterm neonates with a median (interquartile range) GA of 27 weeks (25, 28) and median birthweight of 820 g (710, 900). Scr concentrations were collected up to 6 weeks after birth with a median of 20 observations (14, 25) per patient. In 65%, ELBW neonates were delivered by C-section and 86% received betamethasone prenatally to induce lung maturation. Of our studied population, 64% and 51% of patients received ibuprofen or treatment with inotropic agents, respectively. The evaluation dataset comprised a population with similar median GA and birth weight as the population used for model development (Table 1).

**Table 1:** Demographic data presented as median [interquartile range] or as *n* (%); obs, observations. *Defined as maternal betamethasone treatment before birth

Characteristics	Model building dataset	Evaluation dataset
ELBW neonates	148 (2814)	69 (1212)
obs. per patient	20 [14, 25]	18 [13, 22]
Gestational age (weeks)	27 [25, 28]	27 [26, 28]
Birth weight (g)	820 [710, 900]	840 [735, 910]
Current weight (g)	920 [795, 1105]	924 [786, 1171]
obs. per patient	5 [3, 6]	5 [4, 6]
Ibuprofen treatment (yes)	96 (64%)	37 (54%)
Inotropes treatment (yes)	76 (51%)	23 (34%)
Mode of delivery		
Vaginal	51 (34%)	19 (28%)
Cesarean	97 (65%)	50 (72%)
Sex		
Female	85 (57%)	30 (44%)
Male	63 (42%)	39 (56%)
Lung maturation*	128 (86%)	56 (81%)
Neonatal death	18 (12%)	10 (14.5%)
Crea_conc_ 1st day of life (mg/dl)	0.57 [0.5, 0.68]	0.71 [0.59, 0.83]

### Model development

Data was described by a turn-over model, assuming a constant creatinine production and time-varying (i.e. postnatal age-dependent) net creatinine clearance (baseline clearance (CL_BL_) + postnatal age-dependent clearance increase due to postulated decrease in reabsorption). Hence, if the produced creatinine amount per time interval is greater than the eliminated amount, an increase in Scr will be observed (and vice versa); steady-state Scr will be achieved if the produced creatinine amount equals the eliminated amount.

2 $$ \mathrm{Crea}(0)={\mathrm{Crea}}_{\mathrm{birth}} $$

3 $$ \frac{d\ \mathrm{Crea}}{dt}={\mathrm{kin}}_{\mathrm{production}}-\mathrm{kou}{(t)}_{\mathrm{elimination}}\times \mathrm{Crea}(t) $$

4 $$ \mathrm{kou}{(t)}_{\mathrm{elimination}}=\frac{\mathrm{CL}(t)}{\mathrm{Vd}}=\frac{\left({\mathrm{CL}}_{\mathrm{BL}}+\frac{\mathrm{emax}\times {t}^{\mathrm{Hill}}}{{t_{50}}^{\mathrm{Hill}}+{t}^{\mathrm{Hill}}}\ \right)}{0.7_{\left(\frac{\mathrm{L}}{\mathrm{kg}}\right)}\times {\mathrm{weight}}_{\left(\mathrm{kg}\right)}} $$

The initial condition at time zero was estimated with the parameter Crea_birth_, which represents creatinine concentration at birth (Eq. 2). Input and output rates were defined as the production rate of creatinine (kin_production_, mg/day) and time varying rate constant of creatinine elimination (kou(*t*)_elimination_, day^−1^), which was parameterized in terms of time-dependent creatinine clearance (CL(*t*), L/day) and weight-normalized Vd (Eqs. 3 and 4), where *t* is the time after birth, i.e. postnatal age (day), CL_BL_ corresponds to the baseline creatinine clearance (L/day), emax is the maximum additional achieved clearance (L/day), *t*_50_ reflects the time point (day) where half of emax is achieved (which may be partly interpreted as time point at which creatinine reabsorption decreased by 50%), and the Hill coefficient determines the steepness of the clearance-time relationship. To illustrate the driving force of Scr profiles (Scr production versus elimination), the ratio of the amount of creatinine produced (kin_production_) versus the amount eliminated (kou(*t*)_elimination_ × Scr(*t*)) was calculated for each time point. Sensitivity analysis of Vd did not show substantial bias in estimation of the key population parameters, as the percentage difference between 0.7 L/kg and varying Vds did not exceed 5%, with the exception of two population parameters (kin_production_ and emax; Online Resource 2). Comparing the equation for calculation of total body water suggested by Shaffer et al. (mean (min − max), 0.618 L (0.264–1.25 L) and our 0.7 L/kg (mean (min − max), 0.627 L (0.26–1.28 L)) resulted in a minor difference of 8.7 ml on average [14].

**Fig. 2:**
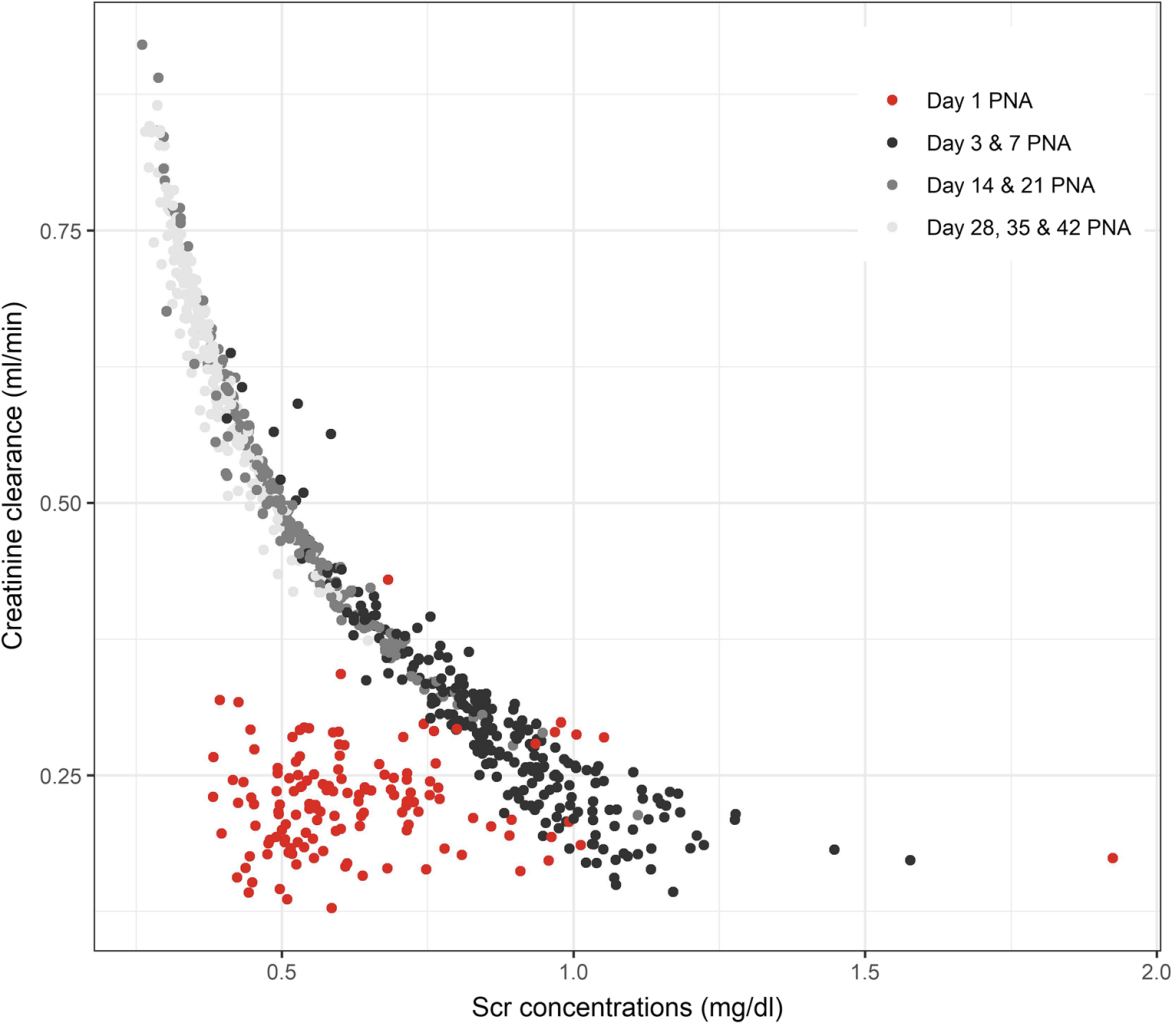
Individual predicted creatinine clearance versus predicted serum creatinine (Scr) concentrations, stratified by postnatal age (PNA)

Inter-individual variability (IIV) could be estimated for the parameters Crea_birth_, CL_BL_, *t*_50_, and Hill (Table 2). In the final model, typical creatinine concentration at birth (Crea_birth_) was estimated to be 0.597 mg/dl with 25.6% IIV and to increase in more mature ELBW cases (+ 6% per increasing week of GA; Table 2). For the typical patient, ibuprofen treatment (yes/no per day) accounted for a 5% decrease (for ibuprofen treatment) in CL_BL_ (25.9% IIV; Fig. 4). The *t*_50_ estimate for a typical patient was 21.1 days and decreased in more mature patients (− 8% per increasing week of GA; shorter *t*_50_ in more mature patients). Additionally, MOD accounted for shorter *t*_50_ when delivered by C-section (− 24%; Table 2). The Hill factor was estimated at 1.31 with 51% IIV. RSEs of the typical population parameters and corresponding IIV values were less than 10%, and RSEs for the covariate-parameter relationships did not exceed 47% (Table 2). No model misspecification was observed as the distribution of the observations was symmetrical around predicted concentrations and the individual weighted residuals are randomly scattered around zero. Predicted creatinine clearance versus Scr concentrations is illustrated in Fig. 2 for different PNA.

**Table 2:** Parameter estimates of final creatinine model together with effect size estimates. CV, coefficient of variation; MOD, mode of delivery (1 for C-section and 0 for vaginal delivery); IBU, ibuprofen treatment (1 for yes and 0 for no treatment). Median GA was set at 26.73 weeks

Parameter (unit)	Estimates (RSE%)	IIV [CV%]
Population parameters		
Crea_birth_ (mg/dl)	0.597 (2.31)	0.252 [25.6]
Kin_production_ (mg/day)	3.55 (1.44)	–
CL_BL_ (L/day)	0.075 (3.95)	0.255 [25.9]
Emax (day^−1^)	0.874 (0.36)	–
*t*_50_ (days)	21.1 (8.72)	0.49 [52]
Hill	1.31 (4.79)	0.481 [51]
Covariate parameters		Parameter-covariate relationship
IBU effect on CL_BL_ (no/yes)	2.73 (0.29) / 2.55 (0.61)	${\mathrm{CL}}_{{\mathrm{BL}}_i}={\mathrm{CL}}_{{\mathrm{BL}}_{\mathrm{pop}}}\times \left(1+2.73\right)\kern0.5em /\kern0.5em {\mathrm{CL}}_{{\mathrm{BL}}_{\mathrm{pop}}}\times \left(1+2.55\right)$
GA effect on *t*_50_	− 2.35 (36.3)	$t{50}_i=t{50}_{\mathrm{pop}}\times {\frac{{\mathrm{GA}}_i}{{\mathrm{GA}}_{\mathrm{m}}}}^{-2.35}\times \left(1-0.24\times \mathrm{MOD}\right)$
MOD effect on *t*_50_	− 0.24 (47)	
GA effect on Crea_birth_	1.6 (21.6)	${\mathrm{Crea}}_{{\mathrm{birth}}_i}={\mathrm{Crea}}_{{\mathrm{birth}}_{\mathrm{pop}}}\times {\frac{{\mathrm{GA}}_i}{{\mathrm{GA}}_{\mathrm{m}}}}^{1.6}$
Residual variability		
Proportional error	0.11 (1.5)	[11]

### Model evaluation

Good predictive performance with high accuracy (MPE (95% CI) − 0.0028 mg/dl (− 0.0075–0.0019)) was obtained from the external analysis (Table 3). The relative MPE (95% CI) was equal to 0.85% (0.14–1.57), and good precision was observed as the relative RMSE (95% CI) amounted to 12.75% (12.11–13.37).

**Table 3:** Evaluation methods to assess the predictive performance of the developed creatinine model

Evaluation methods	Value [95% CI]	Units
Mean prediction error (MPE)	− 0.0028 [− 0.0075–0.0019]	mg/dl
Relative MPE (RMPE)	0.855 [0.139–1.572]	%
Mean squared error (MSE)	0.0069 [0.0061–0.0077]	mg/dl
Relative root mean square error (RMSE)	12.755 [12.108–13.371]	%

### Development of Scr reference ranges: model-based simulations

**Fig. 3:**
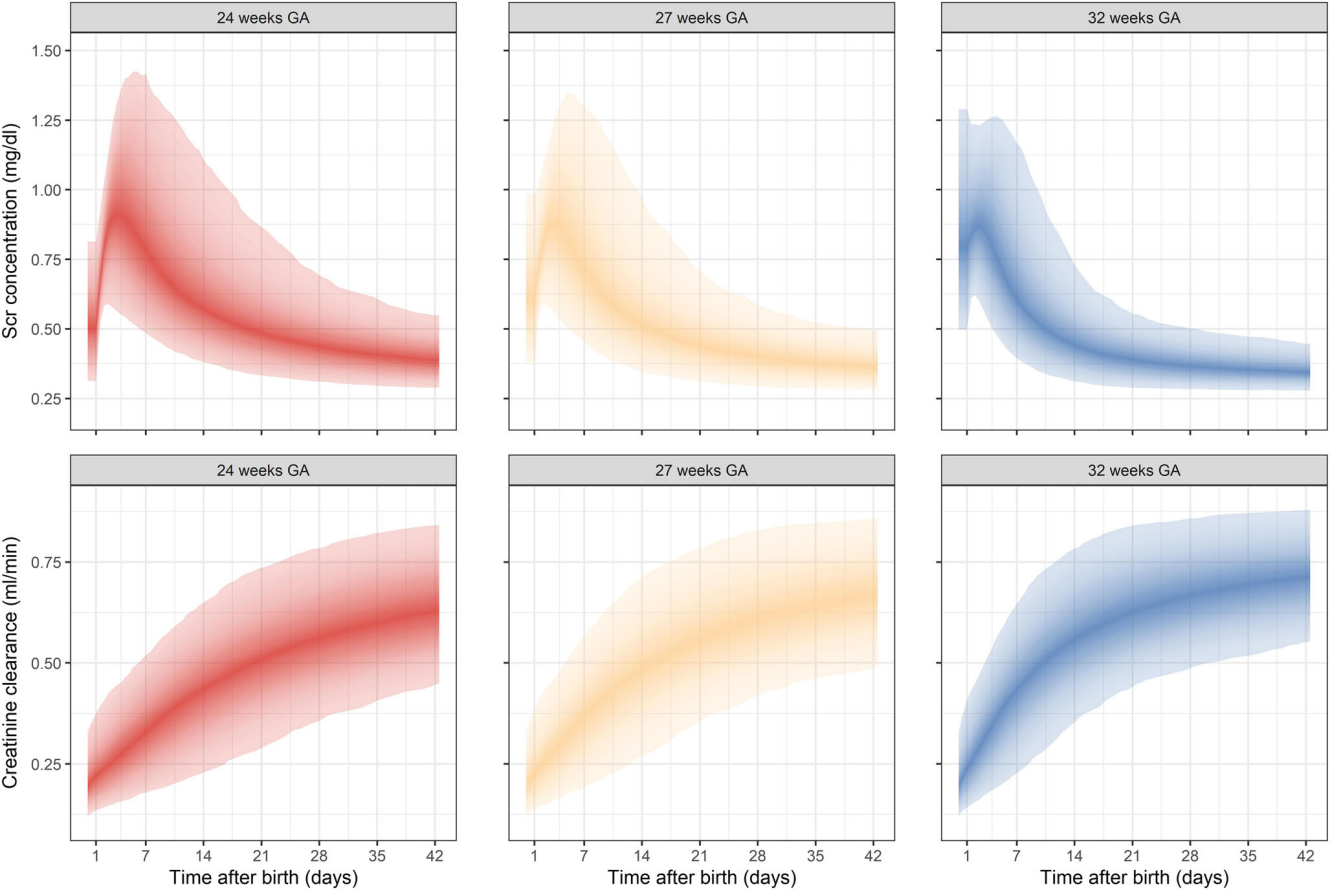
Simulated serum creatinine (Scr) concentration and clearance values for ELBW neonates with inter-individual variability (IIV) and 95% prediction intervals. Upper panel: Scr concentrations for ELBW neonates born at 24, 27, and 32 weeks of gestation. Lower panel: creatinine clearance for ELBW neonates born at 24, 27, and 32 weeks of gestation

Reference ranges for Scr concentrations and creatinine clearance are shown in Fig. 3 and Table 4. Scr concentrations and creatinine clearance values for typical ELBW neonates (no IIV), stratified for three reference GA values, namely 24, 27, and 32 weeks, are illustrated in Figure S1, S2 (Online Resource 3). BSA-transformed creatinine clearances (in ml/min/1.73 m^2^) are presented in Online Resource 4. The ratio of the amount of creatinine produced versus eliminated for typical ELBW neonates, stratified per GA, is illustrated in Figure S3 (Online Resource 3).

**Table 4:** Median [95% prediction interval] values for Scr concentration and creatinine clearance (CLcr) for various postnatal days for three different typical individuals of 24, 27, and 32 weeks of gestation, retrieved from simulations (*n* = 1000). Volume of distribution is based on median weight (Wt) and fixed to 0.7 L/kg

Postnatal age	24 weeks GA			27 weeks GA			32 weeks GA		
	Wt (g)	Scr (mg/dl)	CrCL (ml/min)	Wt (g)	Scr (mg/dl)	CrCL (ml/min)	Wt (g)	Scr (mg/dl)	CrCL (ml/min)
Day 1	621	0.50 [0.31–0.81]	0.22 [0.14–0.37]	779	0.61 [0.38–0.98]	0.23 [0.14–0.39]	889	0.79 [0.50–1.29]	0.24 [0.14–0.41]
Day 3	631	0.90 [0.58–1.24]	0.26 [0.15–0.44]	778	0.87 [0.57–1.19]	0.27 [0.16–0.45]	887	0.86 [0.56–1.24]	0.31 [0.17–0.49]
Day 7	658	0.79 [0.49–1.41]	0.33 [0.18–0.52]	790	0.72 [0.45–1.30]	0.37 [0.19–0.57]	907	0.61 [0.39–1.17]	0.43 [0.23–0.65]
Day 14	724	0.57 [0.38–1.11]	0.44 [0.23–0.65]	854	0.51 [0.35–0.97]	0.48 [0.27–0.71]	1000	0.44 [0.31–0.73]	0.56 [0.35–0.78]
Day 21	795	0.49 [0.33–0.87]	0.51 [0.29–0.74]	935	0.44 [0.31–0.71]	0.56 [0.35–0.78]	1168	0.39 [0.29–0.55]	0.63 [0.44–0.84]
Day 28	882	0.43 [0.31–0.69]	0.56 [0.36–0.78]	1053	0.40 [0.29–0.59]	0.61 [0.42–0.83]	1322	0.36 [0.28–0.50]	0.67 [0.49–0.86]
Day 35	992	0.41 [0.30–0.61]	0.60 [0.41–0.82]	1205	0.38 [0.29–0.52]	0.64 [0.46–0.85]	1434	0.35 [0.28–0.47]	0.69 [0.52–0.87]
Day 42	1132	0.39 [0.29–0.55]	0.63 [0.45–0.84]	1387	0.36 [0.28–0.50]	0.67 [0.49–0.86]	1513	0.34 [0.28–0.45]	0.71 [0.55–0.88]

## Discussion

This is the first study that provides reference ranges for real-world Scr concentrations and associated creatinine clearance for ELBW neonates by characterizing the dynamics of Scr concentrations during the first 6 weeks of life in a quantitative manner by means of a mathematical model. GA, MOD, and ibuprofen treatment were key factors influencing individual creatinine profiles and corresponding creatinine clearance, while prenatal lung maturation and the need for treatment with inotropic agents were not significant covariates. There are several aspects that require further interpretation and context.

First, in 2002, Léger et al. have applied population pharmacokinetic approaches to pediatric creatinine data with the aim of characterizing GFR. Their population comprised children with a mean age of 11 years, making the results not translatable to our preterm neonatal population [7]. Vieux et al. obtained GFR reference values retrieved from regression models based on Scr assessments in plasma and urine on day 7, 14, 21, and 28 of life [4]. Their assessment of median GFR for a 27-week-old neonate on day 14 after birth was higher as compared with our calculations (16.22 ml/min/1.73 m^2^ vs. 9.40 ml/min/1.73 m^2^; Table 4). This difference might be explained by the fact that our population was considered more immature (smaller GA and lower birthweight), and different equations were applied for the calculation of individual BSA (Dubois and Dubois versus Ahn) [16, 18]. In the current study, BSA was calculated based on current weight measurements, reflecting an accurate image of postnatal development. Furthermore, it is well known that bilirubin is one of the most relevant interfering compounds when creatinine is measured. As bilirubin is mainly an issue in early neonatal life, and subsequently disappears, this also indicates that creatinine decrease in absolute values will be more pronounced over the first week(s) of neonatal life, suggested to reflect a higher creatinine clearance. Additionally, different assays were used for the Scr quantification (Jaffe versus enzymatic) [4, 11]. It has been previously shown that Jaffe Scr concentrations were significantly and systematically higher (difference 0.1–0.2 mg/dl) compared with the enzymatic quantification throughout postnatal life [19]. Along the same line, there is a difference of about 0.1 mg/dl in urinary creatinine measurements between a Jaffe and an enzymatic assay [20]. The advantage of our study is that we characterized the dynamics of Scr concentrations over the entire 6 weeks after birth in a quantitative manner rather than comparing the GFR values on separate days in ELBW cases.

Second, controversy remains regarding the first creatinine concentration at birth. We observed lower Crea_birth_ concentrations in the more extreme preterm neonates, who are born during the second trimester (median (95% CI); 0.50 mg/dl (0.31–0.81) at 24 weeks GA; Table 4). ELBW neonates who are born in the third trimester (> 28 weeks) were characterized by higher Crea_birth_ Scr (median (95% CI); 0.79 mg/dl (0.50–1.29) at 32 weeks GA). This trend is also, albeit to a lesser extent, reflected by the maternal creatinine concentrations, which are generally, but marginally lower during the second trimester (0.59 mg/dl) as compared with the third trimester (0.61 mg/dl) [21].

Third, we illustrated a clear relationship between creatinine clearance and Scr concentrations, starting after the first postnatal days (Fig. 2). Adaptation to extra-uterine life or maternal Scr contribution might be potential hypotheses for lack of relationship between creatinine clearance and Scr concentrations on the first day after birth, although this requires further investigation. Overall, it demonstrates that Scr concentrations, which are routinely collected directly after and during the first days after birth, can be interpreted and can provide crucial understanding on the developing kidney function. In addition, we recognize the unit policy was to obtain Scr concentrations frequently. This can be illustrated by comparing our median number of observations (*n* = 20/patient) to a recent reference study, like the Assessment of Worldwide Acute Kidney Epidemiology in Neonates (AWAKEN) study, where the median number of Scr concentrations was ≤ 3 and ≤ 5 per patient, respectively in 10 and 15 of the contributing units [22].

**Fig. 4:**
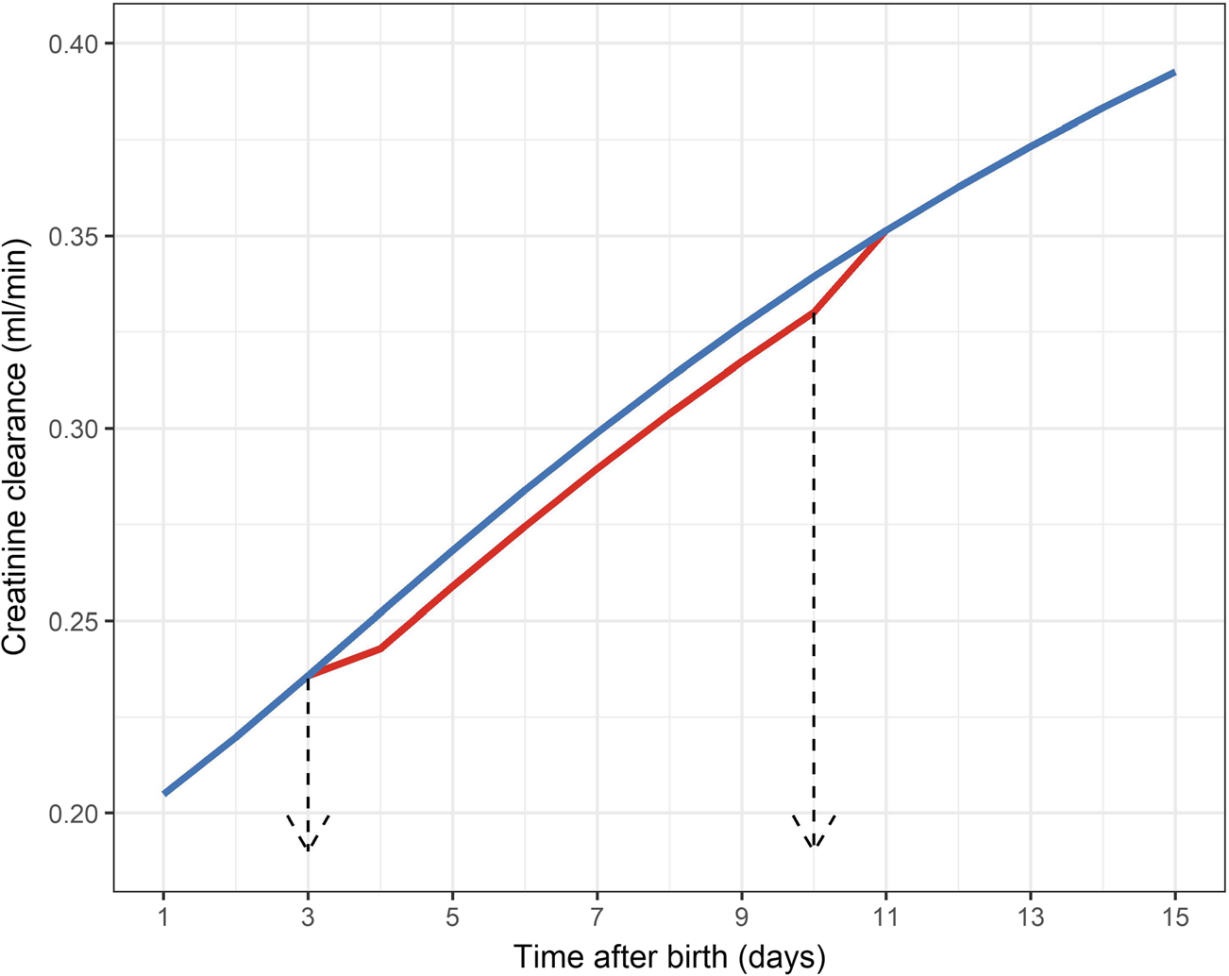
Simulated creatinine clearance of a typical patient of 27 weeks gestational age delivered by C-section receiving 7 days of ibuprofen treatment, starting on third day after birth (red) or without receiving ibuprofen treatment (blue)

Fourth, in this study, antibiotic treatment (e.g. aminoglycosides) has not been assessed as a covariate on any of the model parameters. Since most ELBW neonates in the NICU receive this type of empiric treatment in early neonatal life, we considered this as a nondistinctive feature of our population. The same holds true for nutritional and fluid management strategies. As these interventions are standardized, we were unable to explore these factors. Continuing with drug effects, this study confirms that ibuprofen treatment is associated with a transient decrease in creatinine clearance, although this effect is relatively small (Fig. 4). In contrast, the prescription of inotropic agents was not associated with changes in Scr or creatinine clearance in these datasets. It has to be acknowledged that the dose or exact ibuprofen administration time point during the day is not taken into account; ibuprofen treatment was only assessed as a binary factor on the creatinine clearance for each postnatal day. As such, our analysis may underestimate the effect of ibuprofen treatment on kidney function. Utilized ibuprofen dosing regimen was according to label (10 mg/kg, followed by 5 mg/kg/day every 24 h, for two to four additional days) and was not adapted for the proven increase in ibuprofen clearance with increasing postnatal age [23]. However, we confirm that the model as constructed is indeed fitted to explore potential differences in the incidence and severity of kidney impairment and/or acute kidney injury (AKI) between different dosing strategies.

Fifth, the effect of MOD on Scr concentrations and associated creatinine clearance in our analysis has not been shown before, but co-aligns with the AWAKEN study data, as these authors also observed a positive effect of C-section on the incidence of AKI [22, 24]. It is known that MOD is one of many factors that can influence neonatal physiology, as the recent study by Charlton et al. showed that neonates who were born by scheduled C-section were subjected to a 30% lower probability of acute kidney injury [24]. As acute kidney injury was defined as a Scr increase (0.3 mg/dl), this finding is in accordance with our observations as higher Scr concentrations and lower creatinine clearances were observed for ELBW neonates who were born by vaginal delivery (Figure S2). It has been shown that infants delivered by C-section are subjected to a higher risk for pulmonary hypertension due to the blunted decrease of pulmonary vascular resistance compared with vaginal delivery [25]. Therefore, it might be probable that MOD modulates kidney (function) during early neonatal life since it depends on the vasoconstriction of renal vasculature and its postnatal adaptation pattern, although this hypothesis requires further investigation and more controlled studies. Besides neonatal physiology, maternal morbidities such as preeclampsia and hypertensive disorders are often underlying causes for performing C-section. Whether these maternal morbidities are the cause of lower creatinine concentrations during postnatal life needs to be investigated. However, it is reasonable to anticipate that such maternal morbidities would rather result in higher neonatal Scr concentrations in early neonatal life due to the higher maternal Scr associated with these morbidities [3].

Sixth, our study showed that increasing GA is associated with a steeper increase in GFR over the postnatal period (shorter *t*_50_). No inter-individual variability could be estimated on kin_production_ as it was assumed that the production rate of creatinine remained constant for our ELBW population due to the fact that creatinine is a product that is generated exclusively from muscle metabolism. Previous studies have shown that the fraction of muscle mass does not differ substantially across gestational ages within ELBW cases [26, 27], suggesting that a weight-normalized kin_production_ could have been appropriate. Our estimate of 3.55 mg/day for an average 0.7 L/kg ELBW neonate corresponds to a weight-normalized production rate of 5.1 mg/kg/day, which is approximately half of adult values and can be considered consistent since fractional muscle mass is approximately half that of adults. Additionally, immature kidney tubules in ELBW neonates have poor concentrating ability [28]. As such, these immature and leaky kidney tubules are partially responsible for passive tubular reabsorption of the filtered and accumulated Scr, mostly during the first postnatal days (Figure S3) [10, 29]. The ratio of the amount of creatinine produced versus eliminated over time conceptually illustrates that not acute postnatal kidney function decrease but simply low clearance relative to creatinine production is responsible for the initial rise in Scr after birth. Postnatal water volume constriction might be another contributing factor to this transient increase in Scr, although repeated weight measurements in the first days of life in ELBW cases are uncommon in clinical practice.

Interestingly, a high ratio > 1 (indicating higher amount of creatinine produced than eliminated) was observed during the first 3 days despite constant production of creatinine and increase in clearance of creatinine after birth. It may be hypothesized that this observation is linked to reabsorption of creatinine by immature kidney tubules and that both postnatal (time after birth) and prenatal (gestational age) maturation reduce the reabsorption capability of these tubules. Findings (or results) in our study indicate that GA is an additional factor associated with this ratio, meaning that the amount of creatinine that is being reabsorbed differs per GA.

Finally, especially in a population where the blood volume available for sampling is limited and the risk of anemia is high, restrained management should be pursued when collecting blood samples for e.g. Scr determination. The developed reference ranges could help gain insights in normative creatinine profiles in ELBW neonates and assist in the discrimination between whether a change in Scr concentration is either due to developmental physiological aspects (e.g. as a result of GA or increasing PNA) or due to pathophysiological conditions (e.g. asphyxia or sepsis). For instance, an increase in Scr during the first days of life simply reflects that creatinine production is greater than the eliminated amount per day, up to day 2–3 after birth. The decline in Scr afterwards reflects ongoing postnatal maturation. Observing a substantial increase in Scr after birth for an ELBW neonate born at 32 weeks GA could reflect a pathological condition (e.g. acute kidney injury) rather than immaturity of the kidney [30]. Along the same line, the absence of a Scr decrease may reflect impaired kidney function. Although this study is not focusing on acute kidney injury in ELBW neonates, it needs to be acknowledged that physiological events early after birth cause these parameters to change irrespective of acute kidney injury and that neonatal physiology must be considered in a neonatal-specific definition of acute kidney injury [31].

In conclusion, we report the first mathematical model that is able to characterize creatinine dynamics in ELBW neonates during the first 6 weeks of life in a quantitative manner. Three investigated characteristics influence creatinine clearance, namely GA and MOD (influencing maturation of clearance) and treatment with ibuprofen (directly influencing clearance, but not impeding its maturation), while prenatal lung maturation or the need for treatment with inotropic agents were not significant covariates. Gestational age is further the major determinant for the initial creatinine concentration after birth (increasing 6% per week of GA) suggesting gestational-dependent maternal creatinine transfer to the maternal compartment until birth. The model-derived GA-adjusted reference intervals for ELBW neonates provides a rationale for normative Scr concentrations and as such may assist clinicians to further optimize monitoring and treatment decisions in this vulnerable patient population or to assess adverse drug reactions. This study illustrates a physiological approach to establish population-specific reference ranges, as neonatal physiology is distinct from that of older infants, and suggests a methodological approach to attain this.

## Electronic supplementary material

ESM 1 (DOCX 17 kb)

ESM 2 (DOCX 15 kb)

ESM 3 (DOCX 519 kb)

ESM 4 (DOCX 147 kb)

## Data Availability

Not applicable.

## Abbreviations

BSA: Body surface area
CL_BL_: Baseline creatinine clearance
crea_birth_: Creatinine concentration at birth
ELBW: Extremely low birth weight
GA: Gestational age
GFR: Glomerular filtration rate
IBU: Ibuprofen treatment
IIV: Inter-individual variability
MPE: Mean prediction error
MSE: Mean squared error
MOD: Mode of delivery
NICU: Neonatal intensive care unit
PNA: Postnatal age
RMPE: Relative MPE
RMSE: Relative root mean square error
RSE: Relative standard error
Scr: Serum creatinine
*t*_50_: Time to decrease reabsorption by 50%
Vd: Volume of distribution
