# Supplementary material for: Characterizing dynamics of serum creatinine and creatinine clearance in extremely low birth weight neonates during the first 6 weeks of life
Source: Pediatr Nephrol. 2020 Sep 17;36(3):649–59. doi: 10.1007/s00467-020-04749-3 (PMC7851041; doi:10.1007/s00467-020-04749-3)
Supplement: Supplementary file 1 — (DOCX 17 kb) [file 467_2020_4749_MOESM1_ESM.docx]

**Title**: Dynamics of serum creatinine and creatinine clearance in extremely low birth weight neonates during the first six weeks of life

**Journal**: Journal of Pediatric Nephrology

**Authors**: Tamara van Donge, Karel Allegaert, Verena Gotta, Anne Smits, Elena Levtchenko, Djalila Mekahli, John van den Anker, Marc Pfister

**Corresponding author:**Tamara van Donge, MSc
Pediatric Pharmacology and Pharmacometrics Research
Universitäts-Kinderspital beider Basel (UKBB)
Spitalstrasse 33, CH-4031 Basel, Switzerland
+41 61 704 12 12
[tamara.vandonge@ukbb.ch](mailto:tamara.vandonge@ukbb.ch)

**Online resource 1:**

**Non-linear mixed effect modeling description**

The population analysis was performed by applying non-linear mixed effect modelling approaches. These models take into account both explained and unexplained variability at inter- and intra-individual levels. Non-linear mixed effect models are characterized in terms of:

1. Fixed effects: this is the population average of the model parameters θ. These parameters are susceptible to various factors, such as physiological characteristics (gestational age, body weight, etc), genetic characteristics, or drug-drug interactions. These last factors are the fixed effect covariates, z_i_.
2. Random effects: this is the part of the variability that is not explained by the above fixed effect and allows quantification of:
   - The *inter-individual* variability (also called the between subject variability), which is the variability between two different individuals. It is expressed by ω^2^, which is the variance of the fixed effect parameter θ. For an individual i,
     $\theta_{i}=\theta\cdot\exp\left( \eta_{i} \right)$, with $\eta_{i}\sim N\left( 0,\omega^{2} \right)$

- The *intra-individual* variability (also called the residual unexplained variability), which is the variability within the same individual over time i.e. between two given moments. It is expressed by σ^2^. For an observation j, the corresponding prediction ŷ for an individual is

$y_{i,j}$ = ŷ + $\varepsilon_{i,j}$, with $\varepsilon_{i,j}\sim N(0,\sigma^{2})$

Thus, the general mixed effects model is written:

$\boldsymbol{y}_{\boldsymbol{i,j}}$ **=** $\boldsymbol{f}\left( \boldsymbol{x}_{\boldsymbol{ij}}\boldsymbol{,}\boldsymbol{\phi}_{\mathbf{i}} \right)\boldsymbol{\times}\boldsymbol{\varepsilon}_{\boldsymbol{i,j}}$

And the parameter model:

$\boldsymbol{\phi}_{\mathbf{i}}$ **= g**$\left( \boldsymbol{z}_{\boldsymbol{i}}\boldsymbol{,}\boldsymbol{\theta} \right)\boldsymbol{+}\boldsymbol{\eta}_{\boldsymbol{i}}$

Where $y_{ij}$ is the j^th^ observation in an individual i, $x_{ij}$ are the design variables for an individual i, $\phi_{i}$ is the vector of model parameters for an individual i, and $\varepsilon_{ij}$ represents the residual error; g is a structural model which is function of fixed effects covariates z_i_, and fixed effects parameters θ; finally, $f$ represents the structural model. In this general model, residual error $\varepsilon_{ij}$ is proportional and is assumed to follow a log-normal distribution with mean 0 and a unit variance σ^2^.

Thereby, three parameters have to be estimated:

- the fixed effect vector: $\theta_{i}$
- the random effect parameter quantifying the residual unknown variability: $\sigma^{2}$
- the random effect parameter quantifying the inter-individual variability: $\Omega$.

**Numerical criteria to evaluate the predictive performance of development model**

Evaluation analysis was performed to evaluate the developed creatinine model. The mean percentage error (MPE), relative MPE (RMPE), mean squared error (MSE) and the relative root mean squared error (RMSE) were calculated to evaluate the accuracy and precision of the model predictions.

1. Prediction error (PE) = ${Pred}_{i} -{Obs}_{i}$
2. Relative prediction error (PE%) = $\frac{{Pred}_{i} - {Obs}_{i}}{{Obs}_{i}} \times100$
3. Mean prediction error (MPE) = $\frac{1}{n} \sum\left( {PE}_{i} \right)$
4. Relative MPE = $\frac{1}{n} \sum{(PE\%}_{i})$
5. Mean squared error (MSE) = $\frac{1}{n} \sum{{(PE}_{i})}^{2}$
6. Relative MSE (RMSE) = $\sqrt{\frac{1}{n} \sum{{(PE\%}_{i})}^{2}}$
