# Supplementary material for: Characterizing dynamics of serum creatinine and creatinine clearance in extremely low birth weight neonates during the first 6 weeks of life
Source: Pediatr Nephrol. 2020 Sep 17;36(3):649–59. doi: 10.1007/s00467-020-04749-3 (PMC7851041; doi:10.1007/s00467-020-04749-3)
Supplement: Supplementary file 2 — (DOCX 15 kb) [file 467_2020_4749_MOESM2_ESM.docx]

**Title**: Dynamics of serum creatinine and creatinine clearance in extremely low birth weight neonates during the first six weeks of life

**Journal**: Journal of Pediatric Nephrology

**Authors**: Tamara van Donge, Karel Allegaert, Verena Gotta, Anne Smits, Elena Levtchenko, Djalila Mekahli, John van den Anker, Marc Pfister

**Corresponding author:**Tamara van Donge, MSc
Pediatric Pharmacology and Pharmacometrics Research
Universitäts-Kinderspital beider Basel (UKBB)
Spitalstrasse 33, CH-4031 Basel, Switzerland
+41 61 704 12 12
[tamara.vandonge@ukbb.ch](mailto:tamara.vandonge@ukbb.ch)

**Online resource 2: Sensitivity analyses**

Sensitivity analysis was performed where different Vds are compared, namely 0.3 L/kg, 0.5 L/kg and 0.8 L/kg. Additionally, results of Vd starting at 0.7 L/kg and decreasing with 10% over the study period are shown. Finally, the percentage difference between the final model and combined model (developed dataset + validation dataset) are illustrated.

| **Model parameters of final**  **developed model**  *(Vd = 0.7 L/kg)* | | **Percentage difference**  **with Vd set at 0.7 L/kg** | | | | **% difference with combined model** |
| --- | --- | --- | --- | --- | --- | --- |
|  |  | *Vd of*  *0.3 L/kg* | *Vd of*  *0.5 L/kg* | *Vd of*  *0.8 L/kg* | *10% decrease over PNA* |  |
| **Population parameters** | |  | | | |  |
| Crea_birth_ (mg/dl) | 0.597 | 0.2 | 0 | 0.2 | -0.2 | 6.37 |
| Kin_production_ (mg/day) | 3.55 | -58.3 | -26.8 | 13.2 | 1.9 | -17.5 |
| CL_BL_ (L/day) | 0.075 | -5.9 | -1.1 | -2.7 | 2.52 | -4.9 |
| Emax (day^-1^) | 0.874 | -57.7 | -27.0 | 14.4 | 1.37 | -2.1 |
| t_50_ (days) | 21.1 | 1.4 | 3.3 | 1.4 | 3.2 | 1.9 |
| Hill | 1.31 | -3.0 | 0.8 | -1.5 | 2.29 | 1.5 |
| **Inter-individual variability** | |  | | | |  |
| Crea_birth_ | 0.252 | -1.2 | -0.4 | -1.6 | -0.8 | 1.6 |
| CL_BL_ | 0.255 | 3.9 | -4.3 | 5.9 | -8.6 | 10.2 |
| t_50_ | 0.49 | -1.4 | -1.2 | -0.8 | -0.6 | -7.8 |
| Hill | 0.481 | 0 | 0.21 | -0.2 | -1.0 | 9.9 |
| **Covariate parameters** | |  | | | |  |
| IBU effect on CL_BL_ (no/yes) | 2.73 /  2.55 | -76.9/  -79.1 | -32.2/  -34.1 | 21.2/  20.8 | 6.2/  4.7 | 24.9/  20.8 |
| GA effect on t_50_ | -2.35 | 0.8 | -3.4 | 1.7 | 2.1 | 25.9 |
| MOD effect on t_50_ | -0.24 | 0 | 2.1 | -3.3 | -0.4 | 28.0 |
| GA effect on Crea_birth_ | 1.6 | 0.6 | 2.5 | -0.6 | -1.25 | -17.5 |
| **Residual variability** | |  | | | |  |
| Proportional error | 0.11 | 0 | 0 | 0 | 0 | 1.8 |
