# Supplementary material for: Characterizing dynamics of serum creatinine and creatinine clearance in extremely low birth weight neonates during the first 6 weeks of life
Source: Pediatr Nephrol. 2020 Sep 17;36(3):649–59. doi: 10.1007/s00467-020-04749-3 (PMC7851041; doi:10.1007/s00467-020-04749-3)

**Title**: Dynamics of serum creatinine and creatinine clearance in extremely low birth weight neonates during the first six weeks of life

**Journal**: Journal of Pediatric Nephrology

**Authors**: Tamara van Donge, Karel Allegaert, Verena Gotta, Anne Smits, Elena Levtchenko, Djalila Mekahli, John van den Anker, Marc Pfister

**Corresponding author:**Tamara van Donge, MSc
Pediatric Pharmacology and Pharmacometrics Research
Universitäts-Kinderspital beider Basel (UKBB)
Spitalstrasse 33, CH-4031 Basel, Switzerland
+41 61 704 12 12
[tamara.vandonge@ukbb.ch](mailto:tamara.vandonge@ukbb.ch)

**Online resource 3: Simulated creatinine and creatinine clearance**

Figure S1: Simulated serum creatinine (Scr) concentrations for typical ELBW neonates, stratified per gestational age (GA). Panel A: ELBW neonates delivered by C-section, panel B: ELBW neonates born by vaginal delivery.


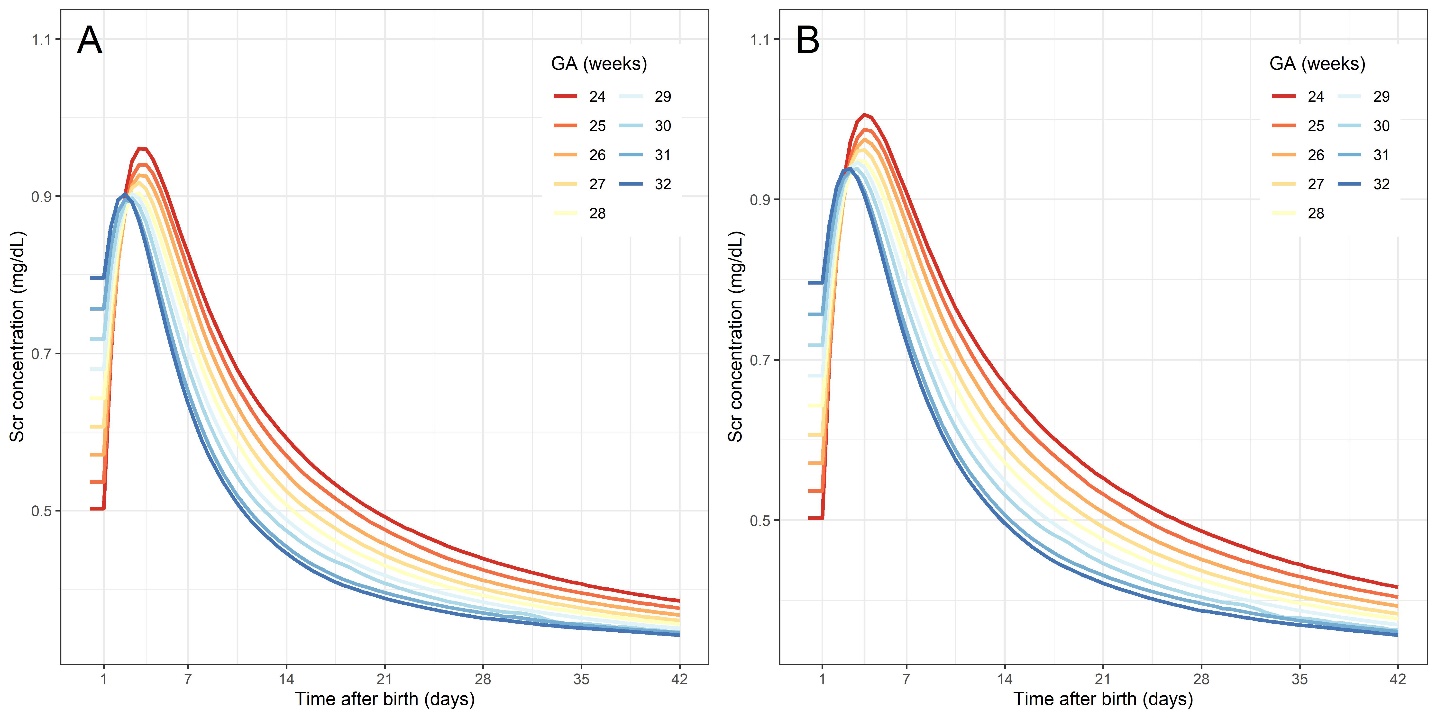


**Figure S2:** Simulated creatinine clearance for typical ELBW neonates, stratified per gestational age (GA). Panel A: ELBW neonates delivered by C-section, panel B: ELBW neonates born by vaginal delivery.


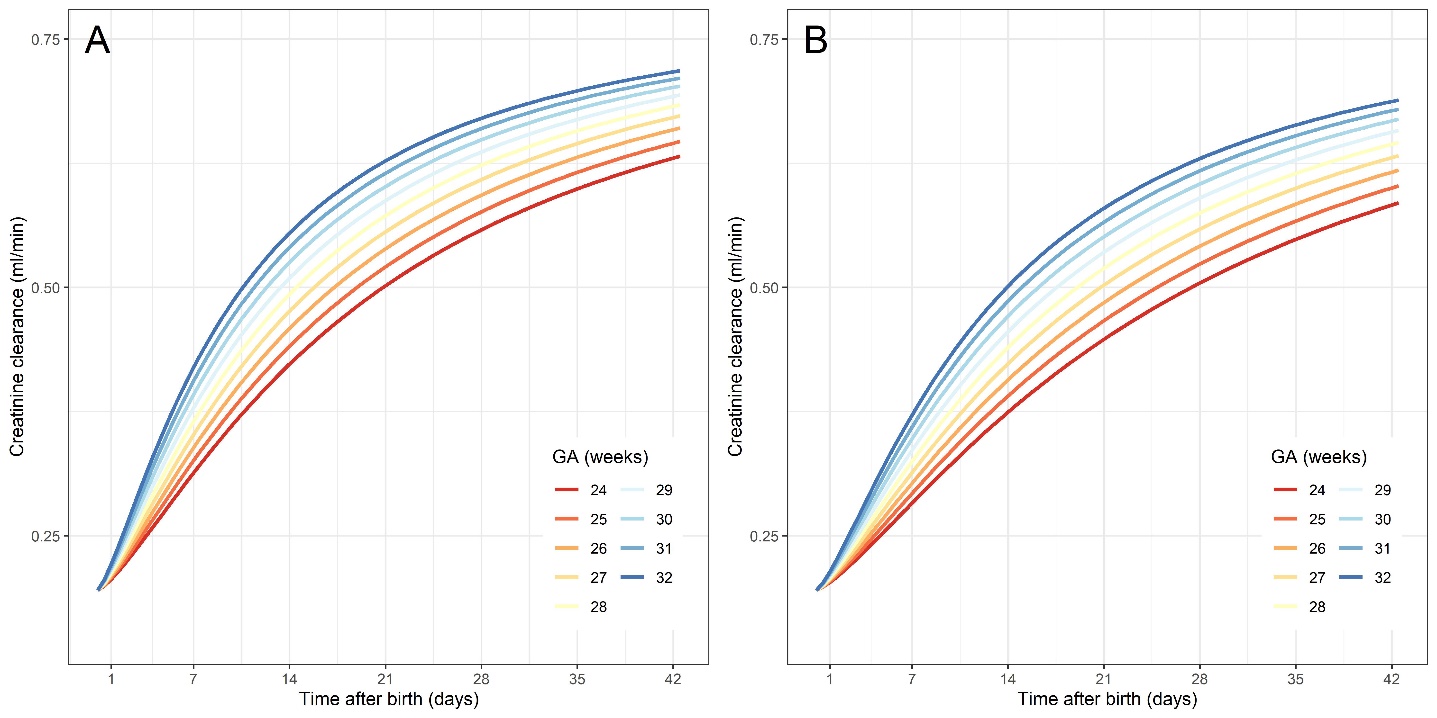


Figure S3: Simulated ratio of creatinine amount (creatinine production-to-elimination) for typical ELBW neonates, stratified per gestational age (GA).


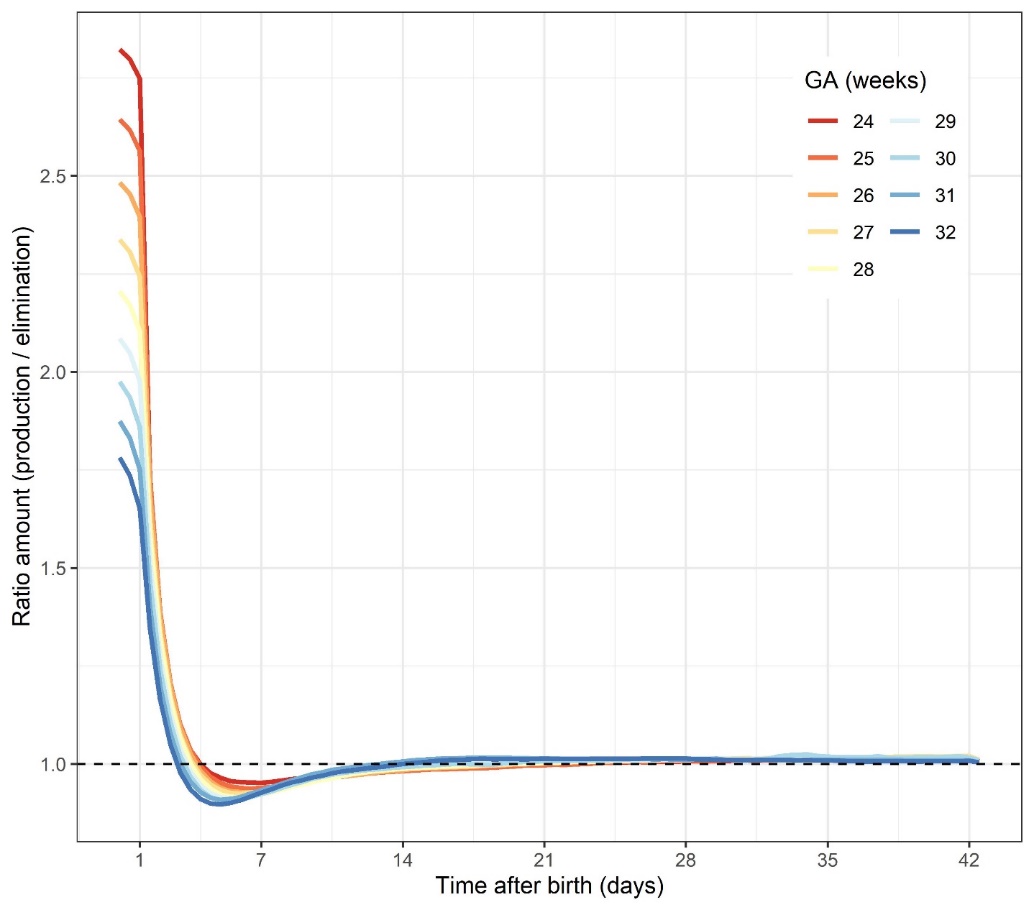

Supplement: Supplementary file 3 — (DOCX 519 kb) [file 467_2020_4749_MOESM3_ESM.docx]
