# Supplementary material for: Characterizing dynamics of serum creatinine and creatinine clearance in extremely low birth weight neonates during the first 6 weeks of life
Source: Pediatr Nephrol. 2020 Sep 17;36(3):649–59. doi: 10.1007/s00467-020-04749-3 (PMC7851041; doi:10.1007/s00467-020-04749-3)

**Title**: Dynamics of serum creatinine and creatinine clearance in extremely low birth weight neonates during the first six weeks of life

**Journal**: Journal of Pediatric Nephrology

**Authors**: Tamara van Donge, Karel Allegaert, Verena Gotta, Anne Smits, Elena Levtchenko, Djalila Mekahli, John van den Anker, Marc Pfister

**Corresponding author:**Tamara van Donge, MSc
Pediatric Pharmacology and Pharmacometrics Research
Universitäts-Kinderspital beider Basel (UKBB)
Spitalstrasse 33, CH-4031 Basel, Switzerland
+41 61 704 12 12
[tamara.vandonge@ukbb.ch](mailto:tamara.vandonge@ukbb.ch)

**Online resource 4: Median [95% prediction interval] values for creatinine clearance per BSA**

Median [95% prediction interval] values for creatinine clearance (CrCL)per body surface area (BSA) for various postnatal days for three different typical individuals of 24, 27 and 32 weeks of gestation, retrieved from simulations (n=1000), Vd is based on median weight. BSA was determined with the formula of Ahn; BSA = 10.602 × weight^0.6561^ / 10000 (BSA in m^2^ and weight in g).

| **Postnatal age** | **24 weeks GA** | | **27 weeks GA** | | **32 weeks GA** | |
| --- | --- | --- | --- | --- | --- | --- |
|  | **Weight**  (g) | **CrCL**  (ml/min/1.73m^2^) | **Weight**  (g) | **CrCL**  (ml/min/1.73m^2^) | **Weight**  (g) | **CrCL**  (ml/min/1.73m2) |
| Day 1 | 621 | 5.29 [3.25 – 8.99] | 779 | 4.68 [2.87 – 8.02] | 889 | 4.53 [2.70 – 7.73] |
| Day 3 | 631 | 6.10 [3.54 – 10.34] | 778 | 5.68 [3.23 – 9.36] | 887 | 5.85 [3.25 – 9.37] |
| Day 7 | 658 | 7.60 [4.13 – 11.97] | 790 | 7.54 [3.94 – 11.64] | 907 | 8.09 [4.25 – 12.09] |
| Day 14 | 724 | 9.45 [4.97 – 14.08] | 854 | 9.40 [5.26 – 13.89] | 1000 | 9.83 [6.21 – 13.75] |
| Day 21 | 795 | 10.35 [5.90 – 15.01] | 935 | 10.30 [6.51 – 14.38] | 1168 | 9.91 [6.99 – 13.31] |
| Day 28 | 882 | 10.72 [6.80 – 14.95] | 1053 | 10.31 [7.12 – 14.06] | 1322 | 9.75 [7.11 – 12.52] |
| Day 35 | 992 | 10.59 [7.19 – 14.51] | 1205 | 9.98 [7.19 – 13.16] | 1434 | 9.63 [7.18 – 12.07] |
| Day 42 | 1132 | 10.17 [7.23 – 13.61] | 1387 | 9.46 [6.91 – 12.14] | 1513 | 9.52 [7.38 –11.75] |


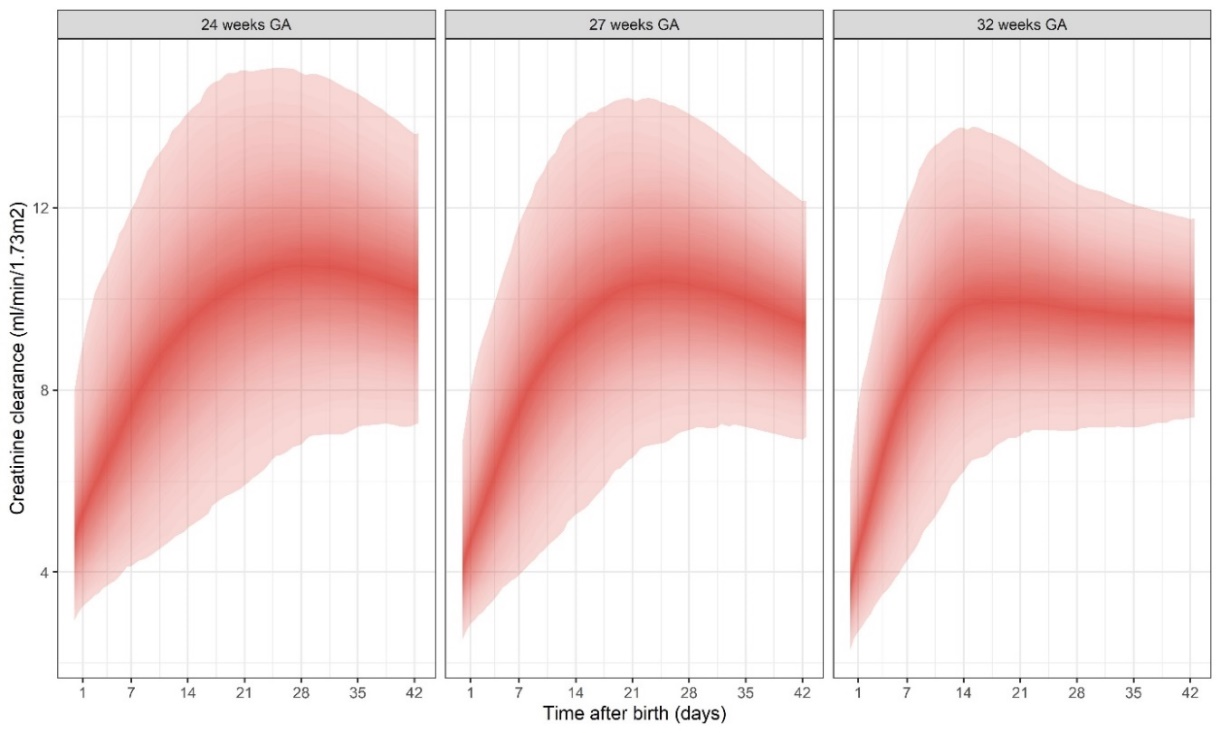

Supplement: Supplementary file 4 — (DOCX 147 kb) [file 467_2020_4749_MOESM4_ESM.docx]
